# Epidemiology of *Clostridium difficile* infection in hospitalized adults and the first isolation of *C. difficile* PCR ribotype 027 in central China

**DOI:** 10.1186/s12879-019-3841-6

**Published:** 2019-03-07

**Authors:** Yu Zhou, Liyan Mao, Jing Yu, Qun Lin, Ying Luo, Xuhui Zhu, Ziyong Sun

**Affiliations:** 1 Department of Laboratory Medicine, Zhejiang Provincial People’s Hospital, People’s Hospital of Hangzhou Medical College, Zhejiang, 310014 Hangzhou China; 2 Department of Laboratory Medicine, Tongji Hospital, Tongji Medical College, Huazhong University of Science and Technology, No. 1095 Jiefang Road, Wuhan, 430030 China

**Keywords:** *Clostridium difficile* infection, *Clostridium difficile* ribotype 027, Risk factor, Molecular characterization, Drug resistance

## Abstract

**Background:**

*Clostridium difficile* infection (CDI) is an emerging healthcare problem in the world. The purpose of this study was to perform a systematic epidemiological research of CDI in Tongji hospital, the central of China.

**Methods:**

Stool samples from hospitalized adults suspected of CDI were enrolled. The diagnosis of CDI were based on the combination of clinical symptoms and laboratory results. Clinical features of CDI and non-CDI patients were compared by appropriate statistical tests to determine the risk factors of CDI. Multilocus sequence typing (MLST) was employed for molecular epidemiological analysis. Susceptibility testing and relevant antimicrobial agent resistance genes were performed as well.

**Results:**

From June 2016 to September 2017, 839 hospitalized adults were enrolled. Among them, 107 (12.8%, 107/839) patients were *C. difficile* culture positive, and 73 (8.7%, 73/839) were infected with toxigenic *C. difficile* (TCD), with *tcdA + tcdB+* strains accounting for 90.4% (66/73) and *tcdA-tcdB+* for 9.6% (7/73). Meanwhile, two TCD strains were binary toxin positive and one of them was finally identified as CD027. Severe symptoms were observed in these two cases. Multivariate analysis indicated antibiotic exposure (*p* = 0.001, OR = 5.035) and kidney disease (*p* = 0.015, OR = 8.329) significantly increased the risk of CDI. Phylogenetic tree analysis demonstrated 21 different STs, including one new ST (ST467); and the most dominant type was ST54 (35.6%, 26/73). Multidrug-resistant (MDR) TCD were 53.4% (39/73); resistance to ciprofloxacin, erythromycin, and clindamycin were > 50%. Other antibiotics showed relative efficiency and all strains were susceptible to metronidazole and vancomycin. All moxifloxacin-resistant isolates carried a mutation in *GyrA* (Thr82 → Ile), with one both having mutation in *GyrB* (Ser366 → Ala).

**Conclusions:**

Knowledge of epidemiological information for CDI is limited in China. Our finding indicated *tcdA + tcdB+ C. difficile* strains were the dominant for CDI in our hospital. Significant risk factors for CDI in our setting appeared to be antibiotic exposure and kidney disease. Metronidazole and vancomycin were still effective for CDI. Although no outbreak was observed, the first isolation of CD027 in center China implied the potential spread of this hypervirulent clone. Further studies are needed to enhance our understanding of the epidemiology of CDI in China.

**Electronic supplementary material:**

The online version of this article (10.1186/s12879-019-3841-6) contains supplementary material, which is available to authorized users.

## Background

*Clostridium difficile* infection (CDI), which is caused by toxigenic *C. difficile* (TCD), has been linked to healthcare facility-associated (HCFA) diarrhea since 1977 [1]. The clinical symptoms of CDI vary from asymptomatic carriage to diarrhea or more severe manifestations, such as pseudomembranous colitis, toxic megacolon and even death [2]. Published data suggest a decline in CDI incidence in hospitalized patients after 2009, but the number of cases remains high. An estimated annual incidence of CDI is 453,000 in the United States, 172,000 in Europe, and 18,005 in England [3, 4], while little is known about the prevalence and impact of CDI in China. The widely accepted major risk factors for CDI include old age (≥65 years), antibiotic exposure, prolonged length of hospital stay, comorbidities such as chronic kidney disease, inflammatory bowel disease, immunodeficiency and immunosuppression [5]. However, reports on community-acquired (CA) CDI have increased among young people who lack the traditional risk factors [6]. Meanwhile, clinical practice guidelines of the Society for Healthcare Epidemiology of America (SHEA) and the Infectious Diseases Society of America (IDSA) claim leukocytosis and increased serum creatinine levels are able to reflect the severity of CDI [7]. Results of a previous study which analyzed 70 patients (> 80 years) with CDI indicated that higher white blood cell counts were independently associated with treatment failure [8].

First detected in North America, the hypervirulent strain *C. difficile* ribotype 027 (CD027), which produces toxin A, toxin B and a third unrelated binary toxin (CDT), as well as carrying an 18 bp deletion in *tcdC* gene, has spread rapidly in various countries in Europe [9]. However, the epidemiology of CDI has changed over the past two decades [10]. Although CD027 remains the dominant clone in the United States, it is rarely reported in Asia. This has occurred simultaneously with an increase in other virulent strains globally. Among them, the toxin A negative and toxin B positive (*tcdA − tcdB+*) strain, has received wide attention [11, 12]. An increasing number of reports mention severe infections and outbreaks caused by *tcdA − tcdB+* strains, with a greater frequency in East Asian countries [13]. Some reports also have shown that *tcdA − tcdB+* strains have significantly higher rates of resistance to clindamycin and moxifloxacin compared with *tcdA + tcdB+* strains [14].

CDI is an emerging problem in Asia, nevertheless, data on CDI in China are limited due to poor clinical awareness. Particularly, the epidemiological distribution, specific risk factors and antimicrobial susceptibility patterns for *C. difficile* isolates are not known well [15]. Genotyping, a useful epidemiological tool, has been widely used for the analysis of evolutionary paths and comparisons of lineages on a global context [16]. A study by Eyre et al. [17] estimated an evolutionary rate of 0.74 SNP per year for *C. difficile* and different molecular characteristics of *C. difficile* has been observed worldwide [2]. Therefore, not only the clinical features and the antibiotic resistance patterns, but also the molecular epidemiology of CDI were investigated in the present study.

## Methods

### Definitions

Diarrhea was defined as more than 3 unformed stools within 24 h (according to Bristol stool chart types 5–7). CDI diagnosis was based on the combination of clinical symptoms and laboratory results, which was defined as the presence of diarrhea and a stool test that was positive for the TCD (Xpert *C. difficile* assay) or clinical evidence of pseudomembranous colitis [7]. Only the first stool sample was collected from each patient. Adult patients were defined as ≥18 years old. The epidemiological associations of CDI were divided into two types: 1) HCFA, the symptoms developed after 48 h of admission or within 12 weeks after discharge from a healthcare facility; 2) CA, the symptoms developed before 48 h of admission and had not been admitted to a healthcare facility in the previous 12 weeks [18]. According to the Public Health Ontario, *C. difficile* outbreaks in healthcare facilities were defined as follows: areas ≥20 beds experiencing three new HCFA *C. difficile* cases within a 7-day period or five new cases within a 4-week period. For units < 20 beds, two new cases in a 7-day period or four new cases within a 4-week period [19].

### Study design and *C. difficile* isolates

From June 2016 to September 2017, hospitalized diarrheal adults who were suspected of CDI by physicians in Tongji hospital (the largest teaching hospital in central China, which treats patients from the six surrounding provinces [20]) were enrolled. Their stool samples (semi-formed, unformed or liquid) submitted to the clinical microbiology laboratory for *C. difficile* detection were collected. The following clinical data of enrolled patients were recorded: demographic data (including age, gender, HCFA versus CA, and comorbidity), presumed risk factors in the 4 weeks before the onset of diarrhea (including prior hospitalization, antibiotic exposure, proton pump inhibitors, nasogastric intubation, abdominal surgery, chemotherapy and immunosuppressive agents treatment), biological parameters (including white blood cell count [WBC], percentage of neutrophile granulocyte [NEU%], hemoglobin [HB], blood platelet count [PLT], albumin [ALB], glutamic oxalacetic transaminase [AST], serum creatinine [CRE] and high sensitivity C reactive protein [hsCRP]), and clinical symptoms (including abdominal pain, fever, vomit and hematochezia) [1]. Relevant laboratory results, only measured within ±3 days of the *C. difficile* detection were recorded. All the collected stool samples were simultaneously subjected to *C. difficile* culture for further study. *C. difficile* colonies were identified on the basis of their typical morphology (flat, black and ground-glass appearance) on selective ChromID *C. difficile* agar (bioMérieux, Marcy l’Etoile, France) and were further confirmed by matrix-assisted laser desorption/ionization time of flight mass spectrometry (MALDI-MS) using the MALDI Biotyper (Bruker Daltonik GmbH, Leipzig, Germany) according to the operating manual [21]. Isolates were stored at − 80 °C using a Microbank® bacterial reservation system (Pro-Lab Diagnostics, Richmond Hill, ON, Canada). This research was approved by the ethics committee of Tongji Hospital. Informed consents were obtained from patients for the use of samples in our research.

### DNA extraction and toxin genes detection

If testing positive for *C.difficile*, genomic DNA of *C. difficile* isolation was extracted from bacterial cultures on blood agar using E.Z.N.A.® Stool DNA Kit (Omega Bio-Tek, Norcross, Georgia, USA) according to the manufacturer’s instructions. The extracted DNA was amplified for the *16 s rDNA*, *tcdA*, *tcdB*, *cdtA*, *cdtB*, and *tcdC* genes of *C. difficile* in a single multiplex PCR, as described in [22]. For the apparently high isolation rate of *tcdA-tcdB+* strains in Asia might reflect mismatching of PCR primers as a result of *C. difficile* polymorphisms [23], all TCD strains were simultaneously detected by NK11/NK9 primer set to verify *tcdA* [24].The standard *C. difficile* strains (ATCC 43596, ATCC 43598, *C. difficile* BI/027/NAP1) obtained from the American Type Culture Collection (Manassas, VA, USA) were used as positive controls for *tcdA + tcdB+*, *tcdA-tcdB+* and binary toxin genes, respectively. *C. difficile* ATCC 700057 (Manassas, VA, USA) was chosen as negative controls for *tcdA*, *tcdB* and the binary toxin genes.

### Multilocus sequence typing (MLST)

Described by Griffiths et al. [25], MLST was used to analyze the sequence types (STs) of all the TCD strains. PCR was conducted to assess seven housekeeping genes (*adk*, *atpA*, *dxr*, *glyA*, *recA*, *sodA* and *tpi*), and the amplicons were further sequenced using forward and reverse primers. The DNA sequences were submitted to the MLST database (http://pubmlst.org/cdifficile/) to obtain the ST types and clade clusters. Based on the tandem sequence of seven housekeeping genes, phylogenic trees were constructed using the neighbor-joining (NJ) method via MEGA software (version 5.2) (http://www.megasoftware.net/). Bootstrapping was performed with 1000 replicates [21].

### PCR ribotyping

PCR ribotyping was performed as a supplement for CD027 identification. Primers 16S (5′-GTGCGGCTGGATCACCTCCT-3′) and 23S (5′-CCCTGCACCCTTAATAAC TTGA CC-3′) were used for classic agarose gel-based PCR ribotyping. Details referred to the methods described by Bidet et al. [26]. *C. difficile* BI/027/NAP1 was used as the reference.

### Susceptibility testing

Minimum inhibitory concentrations (MICs) of 14 antimicrobial agents were determined by the agar dilution method as recommended by the Clinical and Laboratory Standards Institute (CLSI) [27]. Results of antibiotic susceptibility were interpreted according to CLSI guidelines [27] or European Committee on Antimicrobial Susceptibility Testing (EUCAST) [28]. For antimicrobial agents with no standard breakpoints available, resistance was considered as follows: rifampicin, ≥32 μg/ml; fusidic acid, > 0.5 μg/ml; and linezolid, > 4 μg/ml [29]. Strains with resistance to at least three antimicrobial classes were defined as multidrug resistant (MDR). *B. fragilis* ATCC 25285 and *C. difficile* ATCC 700057 were used as quality control strain for susceptibility testing.

### Detection of resistance genes and mutations

For isolates showing resistance to moxifloxacin (MIC ≥8 μg/ml), PCR amplification and sequencing of the quinolone resistance determining region of *GyrA* and *GyrB* were further performed according to the methods reported by Fumie Adachi [30]. Pairwise alignments of DNA sequences were performed using the BLAST server of the National Center for Biotechnology Information.

### Statistical analysis

The results were expressed as medians and quartiles for continuous variables (because most of them were skewed), and as frequencies and percentages for categorical variables. To examine differences of clinical data (including demographic data, presumed risk factors, presumed risk factors, and clinical symptoms) between CDI patients and the non-CDI controls, D-normality test was used for data distribution detection, and Student’s *t*-test was used for continuous data if it was normal distribution, Wilcoxon rank-sum test was employed when the data was abnormal distribution. Chi-square (*χ*2) test was used for categorical data, and if the theoretical frequency of the data in the fourfold table is less than 1, or the total number of cases is less than 40, Fisher’s exact test was employed [10]. Univariate logistic regression analyses were carried out to assess relevant risk factors of CDI. Only statistically different factors were subsequently analyzed in multivariate logistic regression model. False discovery rate (FDR) estimation was used for multiple testing correction. *P* < 0.05 was considered statistically significant. Because antibiotic exposure involves the use of different antibiotics, subgroup analyses of antibiotic were conducted to explore the prevalence of CDI in different situations. Odds ratios (ORs) with 95% confidence intervals (95% CIs) were presented for the logistic regression analyses. SPSS version 18.0 was used for statistical analysis.

## Results

### Clinical features

In total, there were 839 patients suspected of CDI enrolled. In the population studied, males accounted for 70.3% (590/839) and 21.8% (183/839) patients had CA diarrhea. The mean age was 51 (interquartile range: 36–64) years old, with 20.7% (174/839) ≥65 years old. Most patients were admitted in the April–June (41.7%, 350/839), followed by July–September (32.2%, 270/839). 70.4% (591/839) of the patients were from gastroenterology department. Abdominal pain (7.6%, 64/839) and hematochezia (7.5%, 63/839) were the most common symptoms accompanied with diarrhea (details shown in Table 1).

**Table 1:** Basic information and clinical symptoms of the 839 enrolled patients suspected of CDI

Characteristic	Number (%)
Age(years)	
18–30	158 (18.9)
30–40	80 (9.5)
40–50	152 (18.1)
50–65	275 (32.8)
≥ 65	174 (20.7)
Onset of diarrhea	
HCFA	656 (78.2)
CA	183 (21.8)
Sex	
Male	590 (70.3)
Female	249 (29.7)
Season	
Spring (January–March)	114 (13.6)
Summer (April–June)	350 (41.7)
Autumn (July–September)	270 (32.2)
Winter (October–December)	105 (12.5)
Department	
Gastroenterology department	591 (70.4)
Surgery department	49 (5.8)
Hematopathology department	47 (5.6)
Infections department	23 (2.7)
Organ transplantation department	21 (2.5)
Comprehensive medical department	18 (2.1)
ICU	13 (1.6)
Cardiovascular medicine department	13 (1.6)
Respiratory medicine department	13 (1.6)
Other internal medicine department	51 (6.1)
Clinical Symptoms	
Abdominal pain	64 (7.6)
Hematochezia	63 (7.5)
Fever (> 37.2 °C)	40 (4.8)
Vomit	9 (1.1)

*HCFA* healthcare facility-associated, *CA* community-acquired

### Prevalence of CDI and assessment of risk factors

**Fig. 1:**
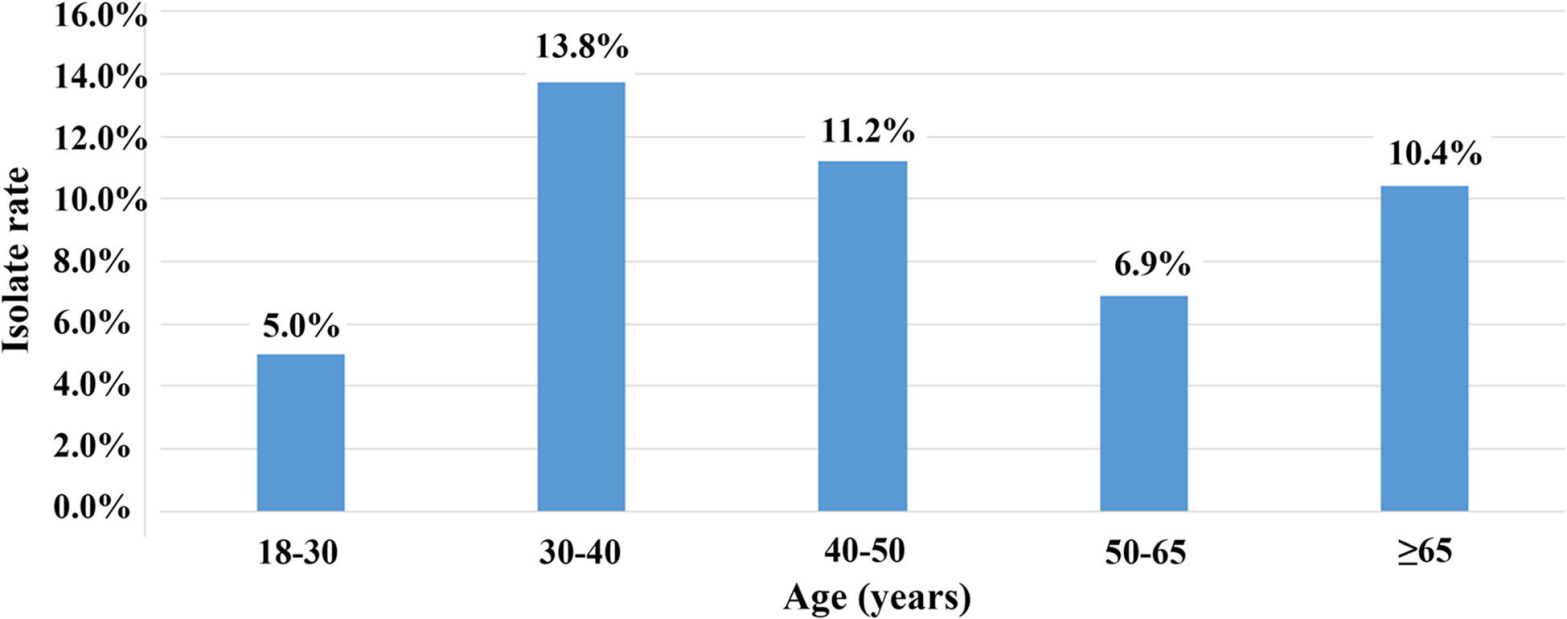
Age distribution of CDI cases (*n* = 73) among enrolled patients (*n* = 839). The isolate rates of different age groups were expressed as percentage

**Fig. 2:**
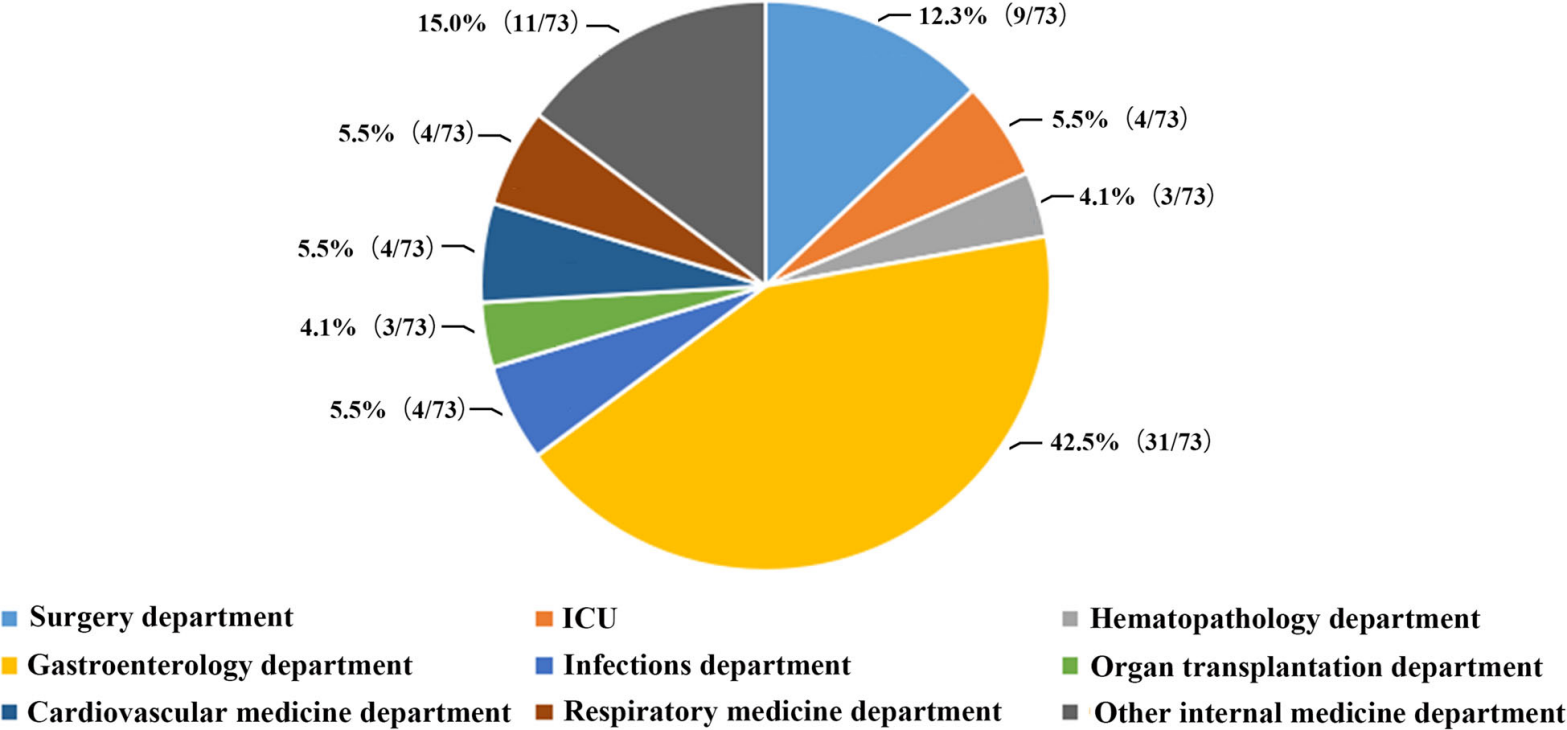
Department distribution of TCD strains (n = 73). The colorful parts of pie chart showed the percentage of TCD distribution in different departments

Among 839 enrolled patients, 107 (12.8%, 107/839) patients were *C. difficile* culture positive and 73 (8.7%, 73/839) TCD strains were isolated. There was a good consistency between the Xpert *C. difficile* assay and multi-PCR in our study, and all the *tcdA-tcdB+* strains were confirmed by two PCR methods. Of the 73 TCD strains, *tcdA + tcdB+* accounted for 90.4% (66/73) and *tcdA-tcdB+* for 9.6% (7/73), with no *tcdA + tcdB-* strains detected. Furthermore, two *tcdA + tcdB+* isolates were positive for binary toxin genes and one of which having deletions in *tcdC* genes was finally identified as CD027 according to the result of GeneXpert, MLST and Ribotyping (gel electrophoresis fingerprint shown in the Additional file 1). Clinical data of those patients with and without CDI were available (shown in Table 2). When age stratification was done, the frequencies of CDI occurring in different age groups were 5.0% (8/159, 18–30 years old), 13.8% (11/80, 30–40 years old), 11.2% (17/152, 40–50 years old), 6.9% (19/275, 50–65 years old), and 10.4% (18/173, ≥65 years old), respectively (Fig. 1). Chi-square test showed no significant difference of CDI incidence between different age groups. Among 73 TCD strains, 31 (42.5%, 31/73) were isolated from the gastroenterology department, 9 (12.3%, 9/73) from surgery department, with ICU, infections department, cardiovascular medicine department and respiratory medicine department sharing the same isolate number 4 (5.5%, 4/73) (Fig. 2).

**Table 2:** Risk factors assessment of 73 CDI patients and 766 non-CDI controls

Characteristic	TCD infection (N = 73) n (%)	Control (*N* = 766) n (%)	*P* value^a^
Demographic data			
Age (Median, Interquartile range)	52 (37.3–64.8)	51 (38–63)	0.908
Age ≥ 65 years	18 (25.0)	155 (20.2)	0.868
Male sex	58 (79.5)	500 (65.3)	0.098
Community-acquired diarrhea	15 (20.5)	168 (21.9)	0.980
Gastrointestinal disease	22 (30.1)	217 (28.3)	1.042
Hepatobiliary disease	6 (8.2)	65 (8.5)	1.026
Cardiovascular disease	6 (8.2)	51 (6.7)	0.910^b^
Kidney disease	9 (12.3)	13 (1.7)	< 0.001 ***
Hemopathy	4 (5.5)	36 (4.7)	1.038^b^
Malignancy	4 (5.5)	21 (2.7)	0.843^b^
Autoimmune disease	1 (1.4)	15 (2.0)	1.000^b^
Diabetes mellitus	5 (6.8)	44 (5.7)	0.922^b^
Presumed risk factors			
Prior hospitalization	43 (58.9)	458 (59.8)	1.030
Antibiotic	53 (72.6)	380 (49.6)	0.003**
No. of antibiotics (≥3)	14 (19.2)	112 (14.6)	0.373
Aminoglycosides	2 (2.7)	10 (1.3)	0.401^b^
β-lactam/β-lactamase inhibitor combinations	34 (46.6)	165 (21.5)	< 0.001***
Carbapenems	17 (23.3)	127 (16.6)	0.365
3rd and 4th generation cephalosporins	3 (4.1)	10 (1.3)	0.320^b^
Fluoroquinolones	24 (32.9)	147 (19.2)	0.030*
Glycopeptide	6 (8.2)	38 (5.0)	0.440^b^
Tetracyclines	3 (4.1)	47 (6.1)	0.681^b^
Metronidazole	4 (5.5)	51 (6.7)	0.697^b^
Vancomycin	4 (5.5)	20 (2.6)	0.296^b^
Proton pump inhibitors	45 (61.6)	421 (55.0)	0.793
Nasogastric tube	11 (15.1)	89 (1.6)	0.842
Abdominal surgery	2 (2.7)	29 (3.8)	1.000^b^
Chemotherapy	2 (2.7)	37 (4.8)	0.947^b^
Immunosuppressive agents	2 (2.7)	10 (1.3)	0.757^b^
Biological parameters			
WBC (×10^9^/L)	6.5 (4.54–9.26)	6.23 (4.89–8.10)	1.034
WBC count > 9.5 × 10^9^/L	17 (23.3)	152 (11.8)	0.939
NEU%	73.6 (64.9–81.4)	64.00 (56.5–75.7)	0.035*
Neutropenia (> 75%)	29 (39.7)	241 (31.5)	0.579
HB (g/L)	116 (92–128)	123.00 (108–135)	0.655
PLT (×10^9^/)	204 (138--271)	192.00 (155–262)	0.933
ALB (g/L)	36 (30.1–41.1)	40.4 (35.3–42.4)	0.420
Hypoalbuminemia (< 35 g/L)	33 (45.2)	206 (26.9)	0.012*
AST (U/L)	19 (13–26)	18 (15–26)	0.999
CRE (umol/L)	73 (53–100)	65 (56–83)	0.728
Serum creatinine > 195 μmol/L	3 (4.1)	10 (1.3)	0.420^b^
hsCRP (mg/L)	11.1 (4.8–77.7)	4.6 (0.7–27.6)	0.426
Clinical symptoms			
Abdominal pain	7 (9.6)	57 (7.4)	0.938
Fever	4 (5.5)	36 (4.7)	0.999^b^
Vomit	1 (1.4)	8 (1.0)	0.982
Hematochezia	6 (8.2)	57 (7.4)	0.978

*WBC* white blood cell count, *NEU* neutrophile granulocyte, *HB* hemoglobin, *PLT* blood platelet count, *ALB* albumin, *AST* glutamic oxalacetic transaminase, *CRE* serum creatinine, *hsCRP* high sensitivity C reactive protein, ^a^False discovery rate (FDR) estimation for multiple testing correction; ^b^Fisher’s exact test; * *p* < 0.05; ** *p* < 0.01; *** *p* < 0.001

Univariate analyses between 73 CDI cases and 766 non-CDI controls were conducted, and the results were shown in Table 2. Kidney disease (*p* < 0.001), antibiotic exposure (*p* = 0.003), NEU% (*p =* 0.035) and hypoalbuminemia (*p* = 0.012) were the parameters found statistically difference between CDI cases and controls. Interestingly, some parameters, such as age, gastrointestinal disease, and proton pump inhibitors, which were commonly considered as risk factors, were found no significant difference. In the multivariate logistic regression model, antibiotic exposure (*p* = 0.001, OR = 5.035) and kidney disease (*p* = 0.015, OR = 8.329) were the factors remained statistically significant (shown in Table 3). Subgroup analyses of antibiotic treatment showed the use of β-lactam/β-lactamase inhibitor combinations (*p* < 0.001) and fluoroquinolones (*p* = 0.030) were the most relevant antibiotics of CDI.

**Table 3:** Results of multivariate logistic regressions for CDI risk factors assessment

Parameters	Multivariate results		
	OR	95%Cl	*P*
Kidney disease	8.329	(1.503, 46.156)	0.015*
Antibiotic exposure	5.035	(1.962, 12.921)	0.001**
NEU%	1.023	(0.997, 1.050)	0.077
Hypoalbuminemia	1.443	(0.676, 3.084)	0.343

*OR* Odds ratios, *95% CI* 95% confidence intervals, * *p* < 0.05; ** *p* < 0.01

### Two cases of CDT+ CDI

There were two CDT+ cases observed, with one of them identified as the hypervirulent strain CD027. The CD027 infection case was an 87 years old man. He was admitted to the respiratory department because of severe pneumonia. Notably, he had diarrhea and fever on the day when he was hospitalized. Referring to his medical history, he had been hospitalized in another hospital 12 days before for the heart stent implantation. Treatment details during that period were unavailable. The biological results showed increased WBC (17.8 × 10^9^ cells/L), NEU% (91.28%) and CRE (109 umol/L), but low level of ALB (26.6 g/L) when *C. difficile* was detected. Another case was a 64 years old woman, who was hospitalized for heart disease and accompanied with multiple organ dysfunction syndrome (MODS). She did not have diarrhea until 25 days after being hospitalized. During this period, she had nasal feeding tube for 3 days; was exposed to meropenem, cefotaxime/sulbatan, moxifloxacin and proton pump inhibitors. When *C. difficile* was detected, the fecal occult blood test was positive. Similar with CD027 infected case, the biological results showed high level of NEU% (87.0%), AST (68 U/L) and CRE (262 umol/L), but low level of ALB (30.9 g/L) when TCD was detected.

### Molecular epidemiology of the TCD isolates

**Fig. 3:**
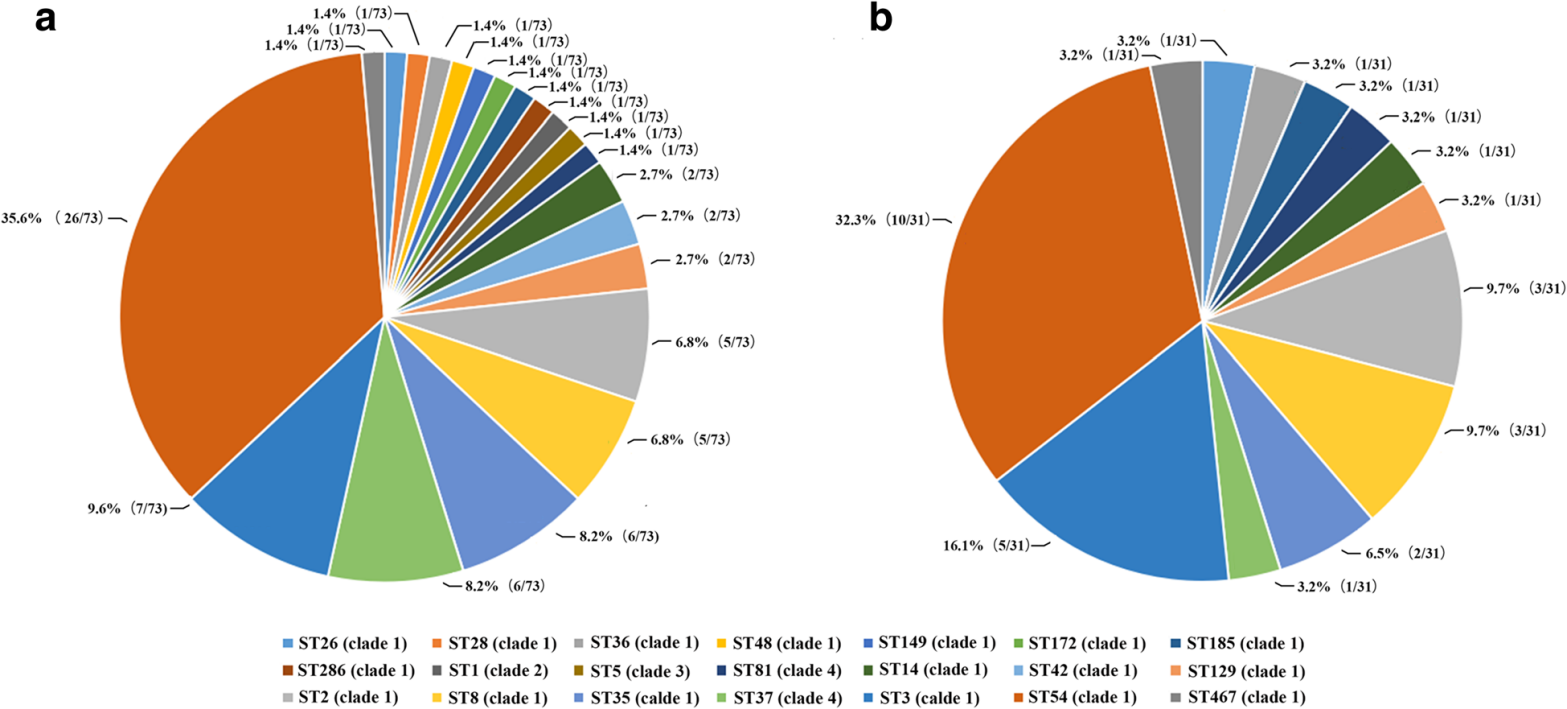
MLST distribution of TCD strains (n = 73). **a** MLST distribution of all isolated TCD strains (n = 73). **b** MLST distribution of TCD strains isolated from the gastroenterology department (*n* = 31). The colorful parts of pie chart showed the percentage of different STs

As shown in Fig. 3, TCD strains analyzed by MLST were assigned to 21 different STs, including one new genotype, ST467 (clade 1). Most genotypes were found to be from clades 1 and 4, and only two CDT+ strains were from clade 2 (ST1) and clade 3 (ST5), respectively. The most prevalent type was ST54 (35.6%, 26/73), followed by ST3 (9.6%, 7/73), ST37 (8.2%, 6/73), ST35 (8.2%, 6/73), ST8 (6.8%, 5/73) and ST2 (6.8%, 5/73). The data indicated a correlation between some STs and the toxin genotypes, e.g. all *tcdA-tcdB+* strains belonged to ST37 (clade 4) except one which belonged to ST81 (clade 4). As most TCD (42.5%, 31/73) were isolated from the gastroenterology department, we specifically analyzed MLST distribution in this department. Overall, 31 TCD strains belonged to 13 STs. Similarly, ST54 was the predominant (32.3%, 10/31) among them, followed by ST3 (16.1%, 5/31), ST8 (9.7%, 3/31) and ST2 (9.7%, 3/31).

### Phylogenetic analysis

**Fig. 4:**
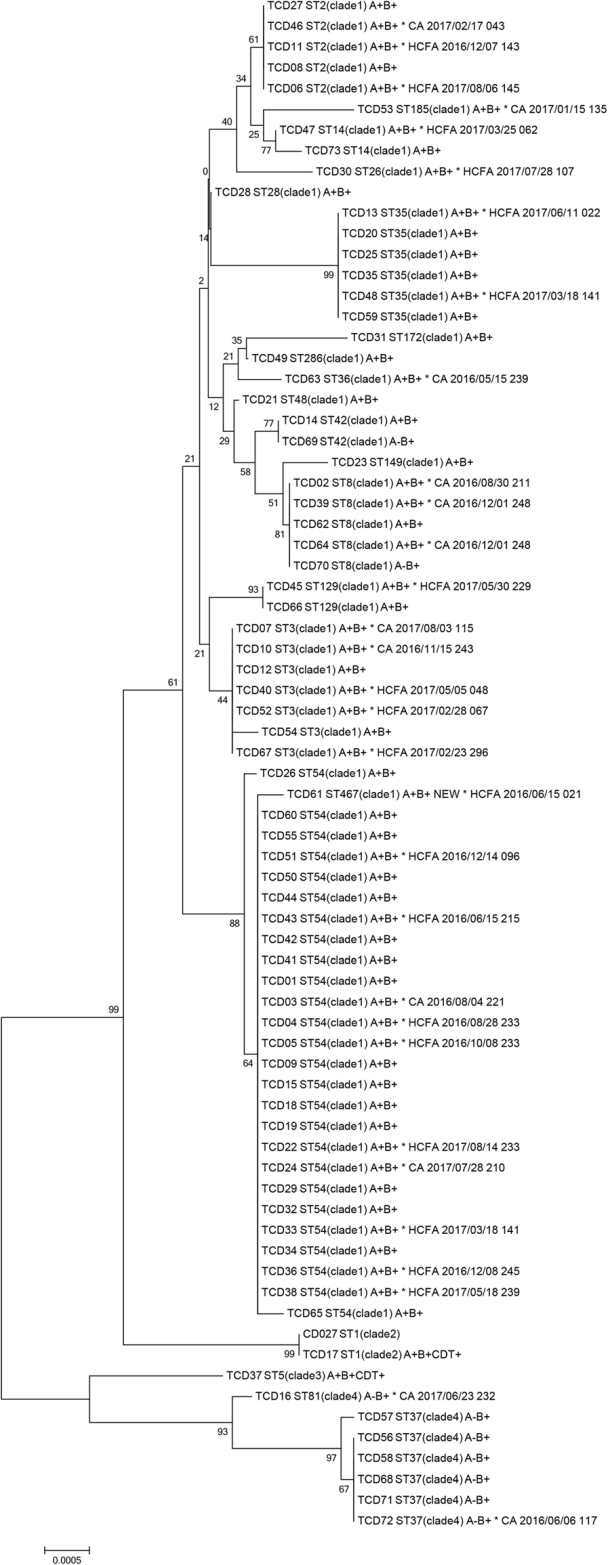
Phylogenetic tree of TCD strains (n = 73). Bootstraps were generated using 1000 replicates and bootstrap values for the cluster were shown on respective branches. ST, clade and toxin types were presented after each TCD strains. For strains isolated from the gastroenterology department, additional information (e.g. onset of diarrhea, admission date and bed number) were presented. CD027 was acted as the reference strain. A, *tcdA*; B, *tcdB*; CDT, binary toxin positive; NEW, new ST type; CA, community-acquired; HCFA, healthcare facility-associated; *, isolated from the gastroenterology department

Phylogenetic analysis showed two major (distinct) lineages (Fig. 4). Strains (11.0%, 8/73) belonging to ST5, ST37 and ST81 constituted one lineage, while the majority (89.0%, 65/73) belonging to other STs were clustered into another lineage. The newly identified ST467 (clade 1) had *glyA* allele changed from allele 1 to allele 5 comparing with ST54 (clade 1). According to the definition of Public Health Ontario, no *C. difficile* outbreak was observed. Relationships between strains isolated from the gastroenterology department were further analyzed. Among 31 TCD isolation, 19 (61.3%, 19/31) cases were classified as HCFA CDI. Interestingly, although no outbreak was detected, three HCFA cases (TCD04, TCD05 and TCD22, all belonging to ST54) were found among patients who occupied the same bed during 2016/08/14 to 2016/10/08.

### Resistance of the TCD strains

The resistant results to 14 antibiotics amongst TCD strains were shown in Table 4 (raw data shown in Additional file 2). The most prevalent resistance was detected for ciprofloxacin (71.2%, 52/73), followed by 61.6% (45/73) for erythromycin, 53.4% (39/73) for clindamycin and 45.2% (33/73) for fusidic acid. Resistance to tetracycline, moxifloxacin and levofloxacin was less common (< 30%). However, all tested isolates were sensitive to metronidazole, piperacillin/tazobactam, vancomycin, rifampicin and linezolid. Among the fluoroquinolones, resistance to ciprofloxacin (71.2%, 52/73) was higher than that of levofloxacin (16.4%, 12/73) and moxifloxacin (12.3%, 9/73). Almost all the moxifloxacin resistant strains (88.9%, 8/9) showed resistant to ciprofloxacin and levofloxacin (seen in the Additional file 2). Meanwhile, MIC values of clindamycin (MIC_90_ > 128μg/ml) and erythromycin (MIC_90_ > 128μg/ml) were rather high. 39 (53.4%, 39/73) TCD isolates were MDR, with two isolates showing intermediate activities to meropenem and chloramphenicol, respectively. As expected, CD027 isolate showed high resistance to fluoroquinolones (ciprofloxacin, MIC> 128 μg/ml; levofloxacin, MIC> 128 μg/ml; and moxifloxacin, MIC = 32 μg/ml). Although the drug susceptibility results of three ST54 strains isolated from patients who once shared the same bed were different, two of them were MDR. Additionally, most of the ST37 strains were sensitive to the antimicrobial agents tested, and only one isolate was MDR.

**Table 4:** MICs of 14 antimicrobial agents for 73 TCD isolates by agar dilution method

Antimicrobial agent	Breakpoint of resistant (ug/ml)	MIC (ug/ml)			No. (%) of isolates		
		MIC_50_ (ug/ml)	MIC_90_ (ug/ml)	Range (ug/ml)	Sensitive	Intermediate	Resistant
Metronidazole	≥ 32	0.25	0.25	≤0.03–1	73 (100)	0 (0)	0 (0)
Piperacillin/tazobactam	≥128/4	4	8	≤0.03–16	73 (100)	0 (0)	0 (0)
Meropenem	≥16	1	2	≤0.03–8	72 (98.6)	1 (1.4)	0 (0)
Tetracycline	≥16	0.25	16	≤0.03–32	54 (74.0)	9 (12.3)	10 (13.7)
Clindamycin	≥8	8	> 128	≤0.03- > 128	27 (37.0)	7 (9.6)	39 (53.4)
Erythromycin	≥8	128	> 128	≤0.03- > 128	28 (38.4)	–	45 (61.6)
Chloramphenicol	≥ 32	4	8	≤0.03–16	72 (98.6)	1 (1.4)	0 (0)
Vancomycin	≥ 32	0.5	1	≤0.03–16	73 (100)	–	0 (0)
Rifampicin	≥ 32	≤0.03	≤0.03	≤0.03–1	73 (100)	–	0 (0)
Levofloxacin	≥8	4	64	≤0.03- > 128	61 (83.6)	–	12 (16.4)
Ciprofloxacin	≥8	8	32	≤0.03- > 128	21 (28.8)	–	52 (71.2)
Moxifloxacin	≥8	2	8	≤0.03–32	64 (87.7)	0 (0)	9 (12.3)
Fusidic acid	> 0.5	0.5	8	≤0.03–16	40 (54.8)	–	33 (45.2)
Linezolid	> 4	2	4	≤0.03–4	73 (100)	–	0 (0)

### Gyrase mutations

Among 53 (72.6%, 53/73) TCD strains showing high resistance to quinolones (MIC ≥8 μg/ml to ciprofloxacin or levofloxacin or moxifloxacin), only 9 (17.0%; 9/53) isolates were moxifloxacin resistant (shown in Table 5). However, sequence analysis demonstrated that all moxifloxacin resistant strains had mutations in *GyrA* (Thr82 → Ile), with one both having mutation in *GyrB* (Ser366 → Ala) (sequencing results shown in Additional file 3).

**Table 5:** The MICs of fluoroquinolones and gyrase mutations for 9 isolates with moxifloxacin resistance

Number of strains	MIC (ug/ml)			Gyrase mutations	
	Ciprofloxacin	Levofloxacin	Moxifloxacin	*GyrA*	*GyrB*
TCD10	0.125	128	16	Thr82Ile	
TCD17	> 128	> 128	32	Thr82Ile	
TCD 27	64	> 128	8	Thr82Ile	
TCD 28	32	16	16	Thr82Ile	
TCD 42	128	> 128	32	Thr82Ile	
TCD 53	8	16	8	Thr82Ile	
TCD 54	64	64	32	Thr82Ile	
TCD 58	128	> 128	32	Thr82Ile	Ser366Ala
TCD 59	64	64	32	Thr82Ile	

## Discussion

CDI has come to prominence in the last decade and is regarded as the leading cause of nosocomial diarrhea among adults in North America and Europe [2]. However, little is known about the epidemiology of CDI in China, especially in its central part [11, 31]. In the present study, 839 adults with suspicion of CDI were evaluated to investigate the prevalence, risk factors, molecular characteristics and antibiotic resistance patterns of CDI. Meanwhile, the first isolation of CD027 in central China was reported.

### Prevalence of CDI and cases of CDT+ isolates

A multicenter study by the Centers for Disease Control (CDC) in the United States revealed the positive rate of CDI ranging from 7 to 20% [32].The prevalence of CDI in our study was 8.7% (73/839), same to Chen et al.’s result [21], but a little lower than another study conducted in eastern China (10.0%) [15]. Searching through the PubMed, systematic epidemiological studies about CDI in Wuhan is extremely rare, the only one founded was Galaydick et al.’s research published in 2015, which indicated the overall prevalence of *C. difficile* was 28% [33]. Although both studies performed PCR method for TCD detection, the demographic data of enrolled patients were different and those who tested positive tended to be older than ours (with a median age of 72 years old VS 52 years old). A meta-analysis showed the pooled incidence of TCD among diarrheal patients in Mainland China was 14%, with a high level of heterogeneity between the estimated rates [31]. This further indicated the influence of different regions in the prevalence of CDI.

*TcdA − tcdB+* strains, most belonging to ST37, has been recognized as a potential epidemic strain in China [12]. Different with other reports [29, 34] but similar with Salazar et al.’s result [35], *tcdA-tcdB+* strains were not common in the present study, only accounting for 9.6% (7/73) of the 73 TCD isolates; and *tcdA + tcdB+* strains, accounting for 90.4% (66/73), was the dominant type. It was noteworthy that two CDT+ isolates were observed in our study, and one of them was finally identified as CD027, which was sparsely reported in China. Both cases infected with CDT+ strains (ST1 and ST5), owned high risk factors of CDI (advanced age, antibiotic exposure, abnormal biological results and accompanied with other sever comorbidities). Since 2003, the first outbreak of CD027 (NAP1/BI/027) reported in North America [36], cases have been reported worldwide. Nevertheless, there was no report in mainland China until Wang et al. [37] identified the first isolation of CD 027 in late 2013. Although, CD027 infected cases have been already described in Hongkong, Beijing and Guangzhou [37–39], this is the first report of CD027 identified in Wuhan, Central China, which indicated the importance to carry out active surveillance for the emergence of hypervirulent *C. difficile*.

### Risk factors of CDI

Practice guidelines indicated the two biggest risk factors of CDI were exposure to antibiotics and the organism [40]; Gualtero et al.’s research [41] demonstrated chronic kidney disease was the most common comorbidities associated with CDI. In concurrence with these studies, the multivariate regression model indicated that prior use of antimicrobial agents (*p* = 0.001, OR = 5.035) and kidney disease (*p* = 0.015, OR = 8.329) were closely related to CDI. To date, several antibiotics have been associated with CDI development in hospitals, such as cephalosporins, clindamycin, penicillins and fluoroquinolones [42]. Among them, quinolones are one of the most common antimicrobial agents used in China, which has been identified as a prominent risk factor for CDI and has been associated with CD027 outbreaks [43]. In agreement with this point, fluoroquinolones (*p* = 0.030) was found increasing the risk of CDI in our study. Additionally, it was observed that use of β-lactam/β-lactamase inhibitor combinations (*p* < 0.001) also associated with CDI, which was consistent with Pakyz et al.’s [44] and Vishwanath et al.’s [45] results. However, Dubberke et al. [46] got the opposite result, which indicated β-lactam/β-lactamase inhibitor combinations were associated with a loss of *C. difficile* colonization. Different situation of antibiotic usage might contribute to different results, which implied additional studies are needed to solve the controversy.

Traditionally, risk factors for CDI include old age (≥65 years), antibiotic use, exposure to healthcare settings and various comorbidities or preexisting conditions [47]. Nonetheless, in the present study, some variables (age, gastrointestinal disease, and proton pump inhibitors) show no significant associations. Jin et al. found the age threshold as a risk factor for CDI patients in eastern China to be lower than the age thresholds seen in developed countries [15]. Meanwhile, reports on CA CDI have increased and revealed alarming trends among young people, who were not at high risk according to the traditional factors [48]. In this study, the mean age of diarrhea patients was only 51 years and 21.8% (183/839) patients had CA diarrhea. Supported by the above views, the difference of demographic distribution might affect the result of logistic regression and explain the reasons for the discrepancy of risk factors.

### Molecular characterization of TCD

Genotyping by MLST identified 21 different STs including one new ST (ST467). This diversity is likely due to the different geographical location of patients hospitalized in our hospital (which treats patients from the six surrounding provinces). Although no isolate belonged to ST11 (usually correlated with hypervirulent ribotype078), one hypervirulent *C. difficile* ST1 (CD027) was observed. Meanwhile, in accord with Chen et al.’s result [21], ST54 (35.6%, 26/73) was the dominant type in our study. Reports from Shanghai [49] and Guangzhou [34] demonstrated all *tcdA-tcdB+* strains were ST37 (clade 4), while a study from Hangzhou found they were assigned to different STs. In the present study, *tcdA-tcdB+* isolates were from two different STs (ST37 and ST81), but most of them (85.7%, 6/7) belonged to ST37.

The novel ST467 (clade 1) was isolated from a male patient in the gastroenterology department. Although phylogenetic analysis suggested that ST467 (clade 1) might evolve from ST54 (clade 1), no similar antimicrobial resistance profiles were observed with ST54 (clade 1) strains isolated from this department (seen in the Additional file 2).

In the present study, most TCD strains (42.5%, 31/73) were isolated from the gastroenterology department. Although no outbreak were observed, there were three HCFA cases (all belonging to ST54) sharing the same bed during two months. As occupying the room of a former patient with CDI contributes to an increased risk of acquiring *C. difficile* [50], effective measures are necessary to prevent the nosocomial transmission of CDI.

### Antimicrobial agents-resistant rate among TCD strains

Antimicrobial resistance of *C. difficile* is highly variable in different populations and countries, ranging from 0 to 100% [51]. Specific antibiotic resistance pattern was also observed in our study. A meta-analysis results which included several studies and a large number of samples in mainland China showed the rates of *C. difficile* resistance to ciprofloxacin, clindamycin and erythromycin are higher than in other counties [31]. In our study, TCD showed highest resistance to ciprofloxacin (71.2%, 52/73), followed by erythromycin (61.6%, 45/73) and clindamycin (53.4%, 39/73), but lower than above results (98.3% for ciprofloxacin, 80.2% for erythromycin, and 81.7% for clindamycin). However, consistent with this study, none of isolated strains were resistant to metronidazole and vancomycin. Metronidazole and vancomycin are the first-line antibiotics for mild to moderate CDI and severe infection, respectively [52]; although resistance to metronidazole and vancomycin is not yet a major issue in china, reduced susceptibility to these antibiotics has been gradually increasing [53], which highlighted the need for constant surveillance. The MDR in our study was similar with Spigaglia et al.’s result (53.4% VS 55%) conducted in European [54], but higher than Putsathit et al.’s (53.4% VS 21.9%) in Thailand [55]. Meeting our expectation, CD027 isolate showed high resistance to fluoroquinolones in the present study. Most ST37 isolates were sensitive to tested antibiotics in our setting. As *tcdA-tcdB+* isolates has been associated with increased antibiotic resistance in many studies [56], further research is needed to solve this discrepancy. A present study indicated the diversity of antimicrobial resistance in these strains [34], therefore, the small number of ST37 isolates in the present study and the regional diversity might be the possible causes.

A recent study showed the resistance rate of moxifloxacin in different regions ranging from 2 to 64% [57]. In the present study, the resistance rate to moxifloxacin was 12.3% (9/73), lower than Chen et al.’ [58] and Jin et al.’s results [15] in China. Resistance to fluoroquinolones is generally caused by two main mechanisms, and the principal mechanism of quinolone resistance in *C. difficile* is determined by mutations in the quinolone resistance-determining region (QRDR) of either DNA gyrase subunit *GyrA* or *GyrB*. Up to now, there are five different amino acid substitutions in *GyrA* (Thr82 → Ile, Thr82 → Val, Asp71 → Val, Asp81 → Asn, and Ala118 → Thr) and six substitutions in *GyrB* (Asp426 → Val, Asp426 → Asn, Arg447 → Leu, Arg447 → Lys, Ser366 → Ala, and Ser416 → Ala) in clinical isolates [59]. Concordance with other studies [58, 59], Thr82 → Ile in *GyrA*, which also characterises the hypervirulent epidemic clone CD027, is the most frequent amino acid change in the present study. Only one strain, showing high resistant to all tested fluoroquinolones, harbored both *GyrA* (Thr82 → Ile) and *GyrB* (Ser366 → Ala). However, Ser366 → Ala is not thought to have a key role in resistance since they are also detected in susceptible strains [51].

### Limitations

This study also had some limitations. First of all, we enrolled all the hospitalized adults when doctor suspected of CDI, which might have differences in physician awareness of CDI between hospitals and caused bias. Meanwhile, we used PCR method for TCD detection and didn’t screened other diarrheal pathogens, which might misidentify *C. difficile* associated and non-CDI associated diarrhea patients. Strictly speaking, CDI is defined as the acute onset of diarrhea with TCD and no other documented cause for diarrhea [7], while many enrolled patients in the present study were CA diarrhea (21.8%, 183/839) and most were admitted in the April–June (41.7%, 350/839), indicating the high-risk of diarrhea caused by other pathogens like rotavirus and salmonella [60, 61]. Therefore, the two step algorithms (e.g. screen with nucleic acid amplification test and then performed Toxin A/B EIA test), currently more recommended methods for rapid diagnosis of CDI [2], should be applied. Secondly, some studies have found that the prevalence of asymptomatic colonization with *C. difficile* is high among adult inpatients [62, 63], while we didn’t detect *C. difficile* in asymptomatic patients and the environment to further explore the possible transmission of CDI. Thirdly, we only studied the *C. difficile* obtained from our setting, a multicenter study should be conducted to represent the characterization of *C. difficile* in central China.

## Conclusion

CDI has generally remained poorly understood in China. The present study carried a systematic epidemiological survey of CDI, and reported the isolation of CD027 for the first time in central China. Several meaningful information were provided here. *TcdA-tcdB+* strains were not the dominant, and most CDI in our setting were caused by *tcdA + tcdB+* strains. Antibiotic exposure and kidney disease were most relevant factors for CDI. Although, reports about reduced susceptibility to common antibiotics has been gradually increasing, up to now, the primary antibacterial agents, metronidazole and vancomycin, are still suitable for empiric treatment against CDI in our hospital. Thr82 → Ile in *GyrA* is the most frequent amino acid change in moxifloxacin resistant isolates. CD027 isolate exhibited high resistant to fluoroquinolones and led to severe symptoms. To our knowledge, it is the first CD027 isolate identified in Hubei China, which deserves our vigilance. Further surveillance is urgent to monitor the emergence of specific highly virulent clones and the antibiotic resistance patterns in China.

## Additional files

Additional file 1: Gel electrophoresis fingerprint of CD027 ribotyping. (TIF 82 kb)

Additional file 2: Raw data of MIC. (XLSX 256 kb)

Additional file 3: Sequencing results for *GyrA* and *GyrB* genes. (DOCX 15 kb)

## Abbreviations

ALB: Albumin
AST: Glutamic oxalacetic transaminase
CA: Community-acquired
CD027: *C. difficile* ribotype 027
CDC: the Centers for Disease Control
CDI: *Clostridium difficile* infection
CDT: Binary toxin
CIs: Confidence intervals
CLSI: Clinical and Laboratory Standards Institute
CRE: Serum creatinine
EUCAST: European Committee on Antimicrobial Susceptibility Testing
HB: hemoglobin
HCFA: Healthcare facility-associated
hsCRP: High sensitivity C reactive protein
MALDI-MS: Matrix-assisted laser desorption/ionization time of flight mass spectrometry
MDR: Multidrug resistant
MICs: Minimum inhibitory concentrations
MLST: Multilocus sequence typing
MODS: Multiple organ dysfunction syndrome
NEU%: Percentage of neutrophile granulocyte
NJ: Neighbor-joining
ORs: Odds ratios
PLT: Blood platelet count
QRDR: Quinolone resistance-determining region
STs: Sequence types
TCD: Toxigenic *C. difficile*
WBC: Blood cell count
