# Supplementary figures and images for: Epidemiology of Clostridium difficile infection in hospitalized adults and the first isolation of C. difficile PCR ribotype 027 in central China
Source: BMC Infect Dis. 2019 Mar 7;19:232. doi: 10.1186/s12879-019-3841-6 (PMC6407249; doi:10.1186/s12879-019-3841-6)

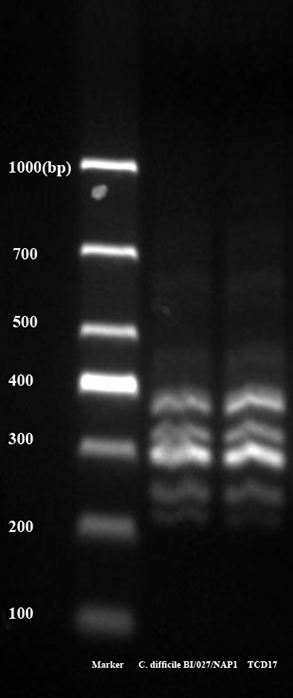

Supplement: Supplementary file 1 — Gel electrophoresis fingerprint of CD027 ribotyping. (TIF 82 kb) [file 12879_2019_3841_MOESM1_ESM.tif]
