# Supplementary material for: Epidemiology of Clostridium difficile infection in hospitalized adults and the first isolation of C. difficile PCR ribotype 027 in central China
Source: BMC Infect Dis. 2019 Mar 7;19:232. doi: 10.1186/s12879-019-3841-6 (PMC6407249; doi:10.1186/s12879-019-3841-6)
Supplement: Supplementary file 3 — Sequencing results for GyrA and GyrB genes. (DOCX 15 kb) [file 12879_2019_3841_MOESM3_ESM.docx]

***GyrA***

**TCD10:**

CAGTTTTGATTATTCATGAGTGTTATAGCTGGACGTGCTCTTCCTGATGTTAGAGATGGTTTAAAGCCAGTTCATAGAAGAATATTATATTCAATGAGTGAGTTAAATTTAACTCCAGATAAACCATACAGGAAGTCAGCTCGTATTGTTGGGGACGTTTTAGGTAAGTACCATCCTCATGGAGATATTGCTGTTTATTATGCTATGGTAAGAATGGCACAAGATTTTTCAACTAGAGCACTTTTAGTAGATGGTCATGGTAACTTTGGTTCTGTTGATGGGGATTCACCAGCTGCTATGCGTTATACAGAAGCTAAAATGAGTAAATTATCATTAGAACTACTAAGAGATATTGAAAAGGAAACTGTAGACTTTAAACCAAACTTTGATGAGTCGTTAAAAGAGCCTTCAGTATTGCCAGCTAGATATCCTAATTTATTAGTAAATGGCTCAAATGGTATAGCTGTTGGTATGGCAACTTCAATACCTCCACATAATTTAGCAGAAGTAATTGATGCAACTGTATATTTGATAGATAATCCAGAGTGTAGTGTAGATGATTTAATAAAATTTGTTCAAGGACCAGATTTCCCAACCAAGGGCTGCAAAA

**TCD17:**

CAGTTTTGATTATTCATGAGTGTTATAGCTGGACGTGCTCTTCCTGATGTTAGAGATGGTTTAAAGCCAGTTCATAGAAGAATATTATATTCAATGAGTGAGTTAAATTTAACTCCAGATAAACCATACAGGAAGTCAGCTCGTATTGTTGGGGACGTTTTAGGTAAGTACCATCCTCATGGAGATATTGCTGTTTATTATGCTATGGTAAGAATGGCACAAGATTTTTCAACTAGAGCACTTTTAGTAGATGGTCATGGTAACTTTGGTTCTGTTGATGGGGATTCACCAGCTGCTATGCGTTATACAGAAGCTAAAATGAGTAAATTATCATTAGAACTACTGAGAGATATTGAAAAGGAAACTGTAGACTTTAAACCAAACTTTGATGAGTCGTTAAAAGAGCCTTCAGTATTGCCAGCTAGATATCCTAATTTATTAGTAAATGGCTCAAATGGTATAGCTGTTGGTATGGCAACTTCAATACCTCCACATAATTTAGCAGAAGTAATTGATGCAACTGTATATTTGATAGATAATCCAGAGTGTAGTGTAGATGATTTAATAAAATTTGTTCAAGGACCAGATTTCCCTAACAAGGCTGCGGAAAA

**TCD27:**

CAATTTTGATTATTCATGAGTGTTATAGCTGGACGTGCTCTTCCTGATGTTAGAGATGGTTTAAAGCCAGTTCATAGAAGAATATTATATTCAATGAGTGAGTTAAATTTAACTCCAGATAAACCATACAGGAAGTCAGCTCGTATTGTTGGGGACGTTTTAGGTAAGTACCATCCTCATGGAGATATTGCTGTTTATTATGCTATGGTAAGAATGGCACAAGATTTTTCAACTAGAGCACTTTTAGTAGATGGTCATGGTAACTTTGGTTCTGTTGATGGGGATTCACCAGCTGCTATGCGTTATACAGAAGCTAAAATGAGTAAATTATCATTAGAACTACTGAGAGATATTGAAAAGGAAACTGTAGACTTTAAACCAAACTTTGATGAGTCGTTAAAAGAGCCTTCAGTATTGCCAGCTAGATATCCTAATTTATTAGTAAATGGCTCAAATGGTATAGCTGTTGGTATGGCAACTTCAATACCTCCACATAATTTAGCAGAAGTAATTGATGCAACTGTATATTTGATAGATAATCCAGAGTGTAGTGTAGATGATTTAATAAAATTTGTTCAAGGACCAGATTTCCTAACACGGCTCTGCCAAAA

**TCD28:**

CATTTTGATTATTCATGAGTGTTATAGCTGGACGTGCTCTTCCTGATGTTAGAGATGGTTTAAAGCCAGTTCATAGAAGAATATTATATTCAATGAGTGAGTTAAATTTAACTCCAGATAAACCATACAGGAAGTCAGCTCGTATTGTTGGGGACGTTTTAGGTAAGTACCATCCTCATGGAGATATTGCTGTTTATTATGCTATGGTAAGAATGGCACAAGATTTTTCAACTAGAGCACTTTTAGTAGATGGTCATGGTAACTTTGGTTCTGTTGATGGGGATTCACCAGCTGCTATGCGTTATACAGAAGCTAAAATGAGTAAATTATCATTAGAACTACTAAGAGATATTGAAAAGGAAACTGTAGACTTTAAACCAAACTTTGATGAGTCGTTAAAAGAGCCTTCAGTATTGCCAGCTAGATATCCTAATTTATTAGTAAATGGCTCAAATGGTATAGCTGTTGGTATGGCAACTTCAATACCTCCACATAATTTAGCAGAAGTAATTGATGCAACTGTATATTTGATAGATAATCCAGAGTGTAGTGTAGATGATTTAATAAAATTTGTTCAAGGACCAGATTTCCCTAAACAAGCTGCAAAACACTC

**TCD42：**

CTTTTTGATATTCATGAGTGTTATAGCTGGACGTGCTCTTCCTGATGTTAGAGATGGTTTAAAGCCAGTTCATAGAAGAATATTATATTCAATGAGTGAGTTAAATTTAACTCCAGATAAACCATACAGGAAGTCAGCTCGTATTGTTGGGGACGTTTTAGGTAAGTACCATCCTCATGGAGATATTGCTGTTTATTATGCTATGGTAAGAATGGCACAAGATTTTTCAACTAGAGCACTTTTAGTAGATGGTCATGGTAACTTTGGTTCTGTTGATGGGGATTCACCAGCTGCTATGCGTTATACAGAAGCTAAAATGAGTAAATTATCATTAGAACTACTGAGAGATATTGAAAAGGAAACTGTAGACTTTAAACCAAACTTTGATGAGTCGTTAAAAGAGCCTTCAGTATTGCCAGCTAGATATCCTAATTTATTAGTAAATGGCTCAAATGGTATAGCTGTTGGTATGGCAACTTCCAATACCTCCACATAATTTTAGCAGAAGTAATTGGATGCAACTGGTATATTTTGATAGATAATCCCAGAGTTGTAGTGTAGATGATTTAATAAAATTTGTTCAGGGACAAAATTTCCTACCAAGGG

**TCD53：**

CAGTTTTGATTATTCATGAGTGTTATAGCTGGACGTGCTCTTCCTGATGTTAGAGATGGTTTAAAGCCAGTTCATAGAAGAATATTATATTCAATGAGTGAGTTAAATTTAACTCCAGATAAACCATACAGGAAGTCAGCTCGTATTGTTGGGGACGTTTTAGGTAAGTACCATCCTCATGGAGATATTGCTGTTTATTATGCTATGGTAAGAATGGCACAAGATTTTTCAACTAGAGCACTTTTAGTAGATGGTCATGGTAACTTTGGTTCTGTTGATGGGGATTCACCAGCTGCTATGCGTTATACAGAAGCTAAAATGAGTAAATTATCATTAGAACTACTGAGAGATATTGAAAAGGAAACTGTAGACTTTAAACCAAACTTTGATGAGTCGTTAAAAGAGCCTTCAGTATTGCCAGCTAGATATCCTAATTTATTAGTAAATGGCTCAAATGGTATAGCTGTTGGTATGGCAACTTCAATACCTCCACATAATTTAGCAGAAGTAATTGATGCAACTGTATATTTGATAGATAATCCAGAGTGTAGTGTAGATGATTTAATAAAATTTGTTCAAGGACCAGATTTCCCTAACAAGGCTGCGGAAAA

**TCD54：**

CTTTTTTGATATTCATGAGTGTTATAGCTGGACGTGCTCTTCCTGATGTTAGAGATGGTTTAAAGCCAGTTCATAGAAGAATATTATATTCAATGAGTGAGTTAAATTTAACTCCAGATAAACCATACAGGAAGTCAGCTCGTATTGTTGGGGACGTTTTAGGTAAGTACCATCCTCATGGAGATATTGCTGTTTATTATGCTATGGTAAGAATGGCACAAGATTTTTCAACTAGAGCACTTTTAGTAGATGGTCATGGTAACTTTGGTTCTGTTGATGGGGATTCACCAGCTGCTATGCGTTATACAGAAGCTAAAATGAGTAAATTATCATTAGAACTACTAAGAGATATTGAAAAGGAAACTGTAGACTTTAAACCAAACTTTGATGAGTCGTTAAAAGAGCCTTCAGTATTGCCAGCTAGATATCCTAATTTATTAGTAAATGGCTCAAATGGTATAGCTGTTGGTATGGCAACTTCAATACCTCCACATAATTTAGCAGAAGTAATTGATGCAACTGTATATTTGATAGATAATCCAGAGTGTAGTGTAGATGATTTAATAAAATTTGTTCAAGGACAAAATTTCCTAACCCAAGGGGTGCGGAAAAAATATAT

**TCD58：**

ATTTAGAATTTGATATTCGATGAGTGTTATAGCTGGACGTGCTCTTCCTGATGTTAGAGATGGTTTAAAGCCAGTTCATAGAAGAATATTATATTCAATGAGTGAGTTAAATTTAACTCCAGATAAACCATACAGGAAGTCAGCTCGTATTGTTGGGGACGTTTTAGGTAAGTACCATCCTCATGGAGATATTGCTGTTTATTATGCTATGGTAAGAATGGCACAAGATTTTTCAACCAGAGCACTTTTAGTAGATGGTCATGGTAACTTTGGTTCTGTTGATGGGGATTCACCAGCTGCTATGCGTTATACAGAAGCTAAAATGAGTAAATTATCATTAGAACTACTGAGAGATATTGAAAAGGAAACTGTAGACTTTAAACCAAACTTTGATGAGTCATTAAAAGAGCCTTCAGTATTGCCAGCTAGATATCCTAATTTATTAGTAAATGGCTCAAATGGTATAGCTGTTGGTATGGCAACTTCAATACCTCCACATAATTTAGCAGAAGTAATTGATGCAACTGTATATTTGATAGATAATCCAGAGTGTAGTGTAGATGATTTAATAAAATTTGTTCAAGGACCAGATTTCCC

**TCD59：**

CATTTTGATATTCATGAGTGTTATAGCTGGACGTGCTCTTCCTGATGTTAGAGATGGTTTAAAGCCAGTTCATAGAAGAATATTATATTCAATGAGTGAGTTAAATTTAACTCCAGATAAACCATACAGGAAGTCAGCTCGTATTGTTGGGGACGTTTTAGGTAAGTACCATCCTCATGGAGATATTGCTGTTTATTATGCTATGGTAAGAATGGCACAAGATTTTTCAACTAGAGCACTTTTAGTAGATGGTCATGGTAACTTTGGTTCTGTTGATGGGGATTCACCAGCTGCTATGCGTTATACAGAAGCTAAAATGAGTAAATTATCATTAGAACTACTGAGAGATATTGAAAAGGAAACTGTAGACTTTAAACCAAACTTTGATGAGTCGTTAAAAGAGCCTTCAGTATTGCCAGCTAGATATCCTAATTTATTAGTAAATGGCTCAAATGGTATAGCTGTTGGTATGGCAACTTCAATACCTCCACATAATTTAGCAGAAGTAATTGATGCAACTGTATATTTGATAGATAATCCAGAGTGTAGTGTAGATGATTTAATAAAATTTGTTCAG

***GyrB***

**TCD58:**

GGGGGCATTTGAGAGTTAGTTGATAGTGTAACAGTTGATGAACTGGGGTCTTTCCTAGAAGAAAACCCAGCAACAGCAAGAATAATAGTTGATAAAGCTTTAAGAGCACAAAGAGCTAGAGAAGCTGCAAAAAAAGCAAGAGAATTAACAAGAAGAAAAAGTGTATTGGAAAGTACATCTTTACCTGGAAAACTTGCAGATTGTGCAGAAAAAGATCCATCTAAAAGTGAAATATTCTTAGTCGAAGGGGATTCAGCGGGAGGTTCAGCTAAACAAGGTAGAGATAGAAATAGTCAAGCTATACTTCCATTAAGAGGTAAAATACTTAATGTTGAGAAATCAAGACTAGATAGGATATTATCTTCAGATGAAATAAAAAATATGATAACAGCTTATGGTTGTGGTATTGGAGAAGATTTTGATATAGATAAGGCTAGGTATCATAAAATTATAATTATGACCGATGCTGATGTAGAATGGGAGCCCCAACCAAAA
